# Supplementary material for: A unified mechanism for innate and learned visual landmark guidance in the insect central complex
Source: PLoS Comput Biol. 2021 Sep 23;17(9):e1009383. doi: 10.1371/journal.pcbi.1009383 (PMC8491911; doi:10.1371/journal.pcbi.1009383)
Supplement: S1 Text — Fig A.Simulations with no modulation of the EPG-PFL synapses.A. 5 examples of paths where the EPG-PFL3 synapse weights are set equal and constant. B. Boxplot (Median: Red, Inter-quartile: shaded box) of the exploration ratio, calculated as the percentage of the 360° surroundings faced by the agent, during 50 simulations. C. Heatmap of 50 simulations stacked adjusted to the cue location (0°). D. Probability density function of the angular speed (all 50 simulations data stacked) corresponding to the 10° s.d. gaussian noise applied to the output of the steering model (red line). Fig B. Innate attraction under the control of a visual reward signal. Simulations of the FB steering model (Fig 6) using a reward signal provided by the visual processes to modify the EPG-PFL synapse weights. We created the visual input signal to the CX using different masks. Results for each panel include (a) the final path directions (n = 50 simulations), (b) the probaility density function of the final direction of the 50 simulstions, (c) the averaged EPG-PFLs synapse weights and (d) examples of 5 simulation paths. A. Visual input to the FBs is equal to the sum of the visual units signal through a continuous proportional mask from 0 (rear units) to 1 (frontal units). B. Visual input to the FBs is equal to the sum of the visual units signal through a continuous proportional mask from -0.5 (rear units) to 0.5 (frontal units). C. Visual input to the FBs is equal to the sum of the visual units signal through a discrete mask equal to 0 (outside the 30° frontal area) or 1 (inside the 30° frontal area). D. Visual input to the FBs is equal to the sum of the visual units signal through a discrete mask equal to 0 (outside the 30° frontal area and the 30° rear area), 0.5 (inside the 30° rear area) or 1 (inside the 30° frontal area). Fig C. Alternative memory model. A. Model diagram. The memory is integrated as a separate set of neurons (similar to CPU4 in [21]), which receive inputs from the reward sig [file pcbi.1009383.s001.pdf]

Supporting information

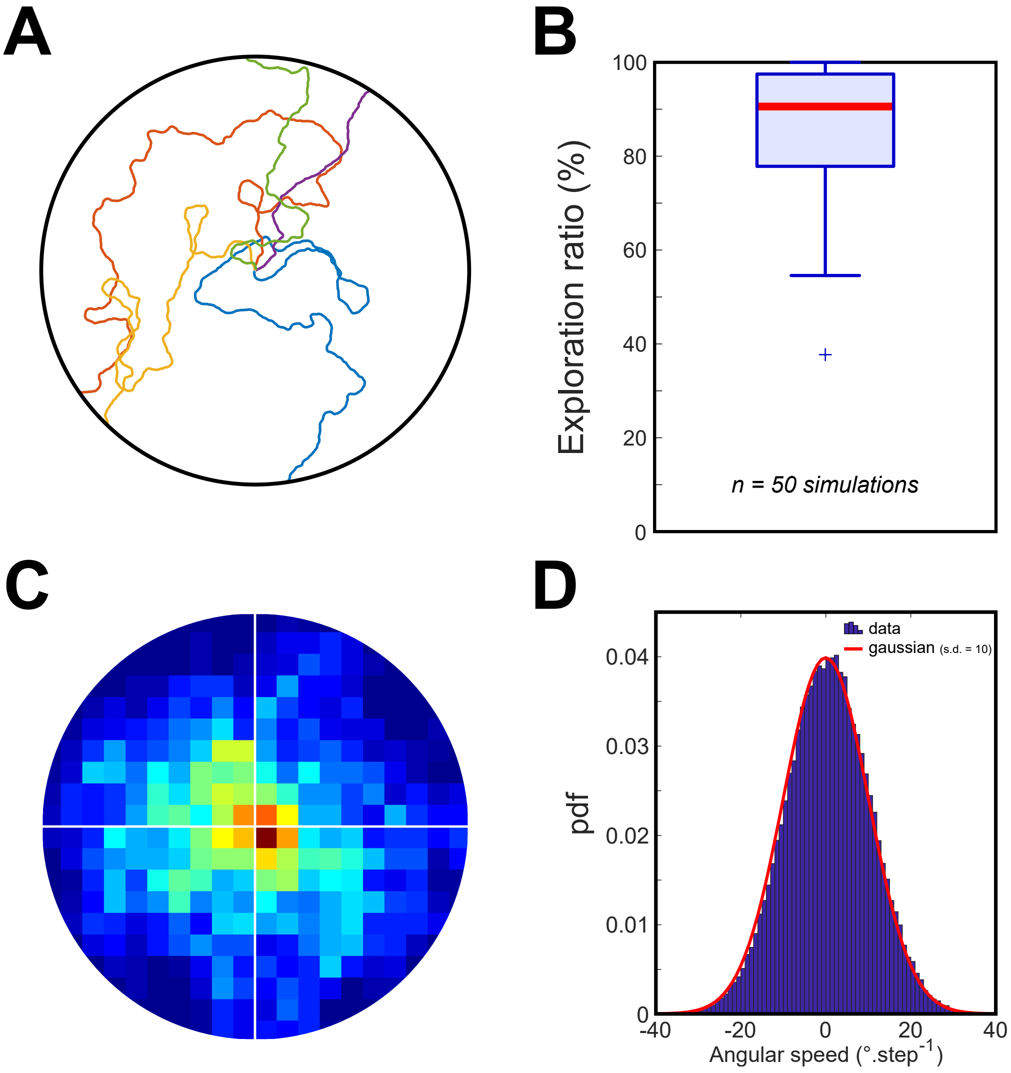

Fig A. Simulations with no modulation of the EPG-PFL synapses.
A. 5 examples of paths where the EPG-PFL3 synapse weights are set equal and constant.
B. Boxplot (Median: Red, Interquartile: shaded box) of the exploration ratio, calculated as the percentage of the 360° surroundings faced by the agent, during 50 simulations.
C. Heatmap of 50 simulations stacked adjusted to the cue location (0°).
D. Probability density function of the angular speed (all 50 simulations data stacked) corresponding to the 10° s.d. gaussian noise applied to the output of the steering model (red line).

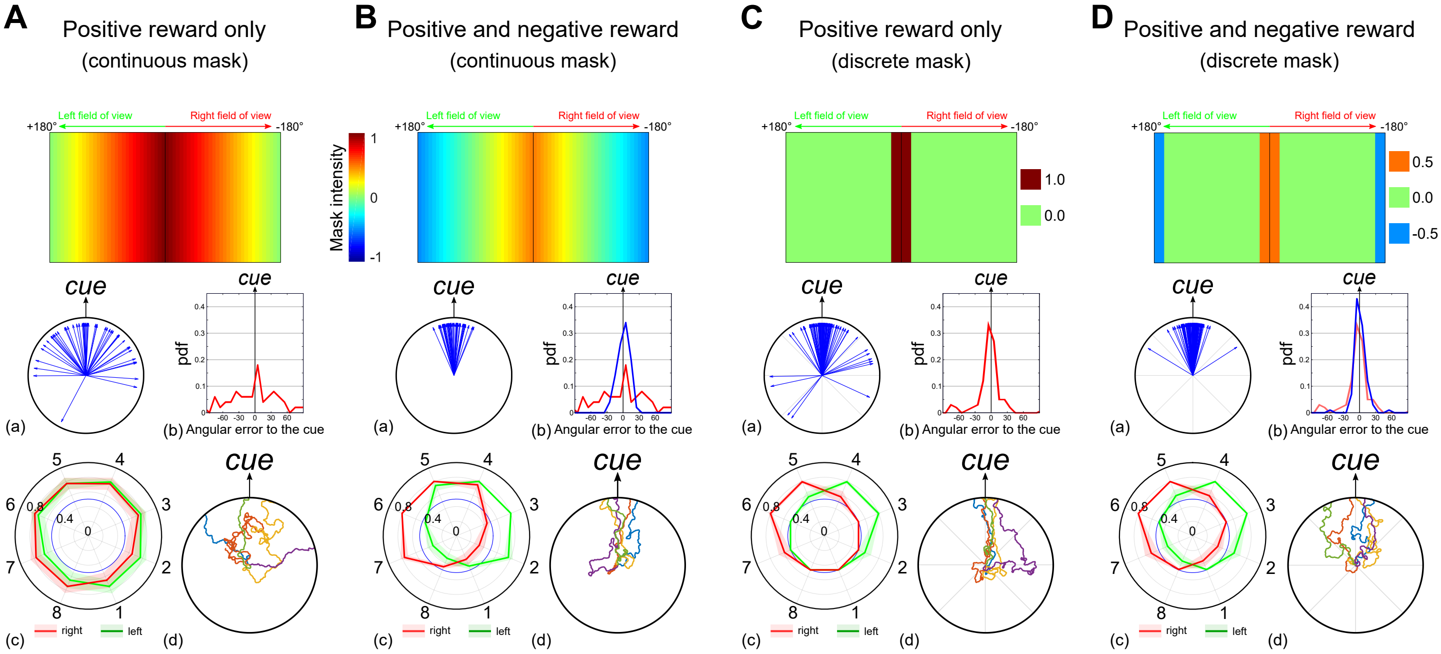

Fig B. Innate attraction under the control of a visual reward signal.
Simulations of the FB steering model (figure 6) using a reward signal provided by the visual processes to modify the EPG-PFL synapse weights. We created the visual input signal to the CX using different masks. Results for each panel include (a) the final path directions (n = 50 simulations), (b) the probaility density function of the final direction of the 50 simulstions, (c) the averaged EPG-PFLs synapse weights and (d) examples of 5 simulation paths.
A. Visual input to the FBs is equal to the sum of the visual units signal through a continuous proportional mask from 0 (rear units) to 1 (frontal units).
B. Visual input to the FBs is equal to the sum of the visual units signal through a continuous proportional mask from -0.5 (rear units) to 0.5 (frontal units).
C. Visual input to the FBs is equal to the sum of the visual units signal through a discrete mask equal to 0 (outside the 30° frontal area) or 1 (inside the 30° frontal area).
D. Visual input to the FBs is equal to the sum of the visual units signal through a discrete mask equal to 0 (outside the 30° frontal area and the 30° rear area), 0.5 (inside the 30° rear area) or 1 (inside the 30° frontal area).

Alternative memory model

In addition to modeling the CX memory based on a synaptic modulation, we tested a model similar to the path integrator memory from [1]. In this case, the input to the CPU4 analog are similar to the previously proposed FBn in addition to the reward signal. Note that the reference here to CPU4 is made for clarity and comparison to [1] and not to support the participation of this particular neuron population to the presented mechanism. The CPU4 population is considered to accumulate activity based on its input. The total input to and the activity level of CPU4 is determined as follow:

$$\begin{cases} I_{CPU4_i^R}(t) &= (EPG_i(t) - NodR(t))\ Rew_{CX}(t) \\ I_{CPU4_i^L}(t) &= (EPG_i(t) - NodL(t))\ Rew_{CX}(t) \end{cases} \quad (1)$$

$$\begin{cases} CPU4_i^R(t) &= CPU4_i^R(t-1) + \alpha\ I_{CPU4_i^R}(t) \\ CPU4_i^L(t) &= CPU4_i^L(t-1) + \alpha\ I_{CPU4_i^L}(t) \end{cases} \quad (2)$$

With $I_{CPU4_i^R}(I_{CPU4_i^L})$ the input to $CPU4_i^R(t)(CPU4_i^L(t))$ and α a free parameter set to modulate the memory acquisition. Then, the PFL3 (or CPU1a) receive excitatory inputs directly from the EPGs and inhibitory inputs from the CPU4, with a column shift as follow:

$$\begin{cases} PFL3_i^R(t) &= EPG_i(t) - CPU4_{i-1}^R(t) \\ PFL3_i^L(t) &= EPG_i(t) - CPU4_{i+1}^L(t) \end{cases} \quad (3)$$

Both encoded vector are therefore compared, the current heading (EPG) and the desired heading (CPU4), and control the steering in the same fashion as proposed in figure 5&6, comparing the summed PFL3 output on the left and on the right. Note that as the CPU4 inputs are inhibitory, the comparison between left and right is opposed to the one from the synaptic modulation memory:

$$\Delta_{steer}(t) \quad = \quad \sum PFL3_i^{left}(t) - \sum PFL3_i^{right}(t) + \epsilon_{steer}^{10} \quad (4)$$

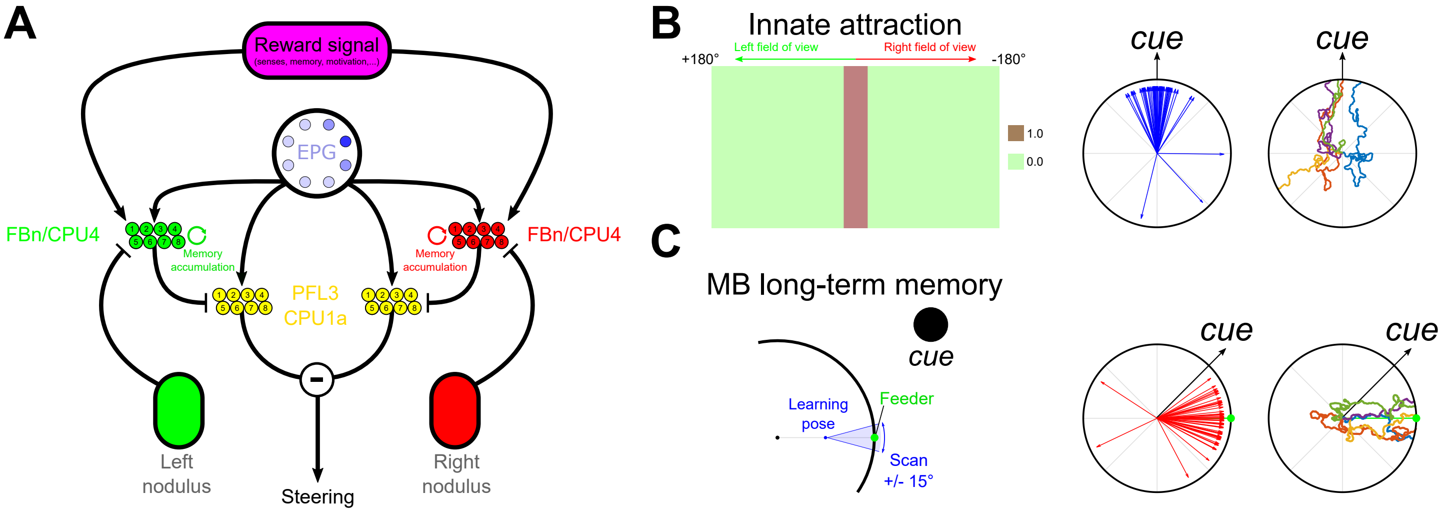

Fig C. Alternative memory model.
A. Model diagram. The memory is integrated as a separate set of neurons (similar to CPU4 in [1]), which receive inputs from the reward signal (Vin or MB), the self-motion and the EPGs. The inhibition from FBns/CPU4s to the PFL3s/CPU1as present the same shift of 1 column as the model described in figure 6.
B. Results from simulation where the reward signal is provided by the visual system and generate an innate attraction to the cue (discrete positive mask).
C. Results from simulation where the reward signal is provided by the MBs after an initial learning phase. In this case the MB is a singular structure providing a similar signal to each side of the CX model.

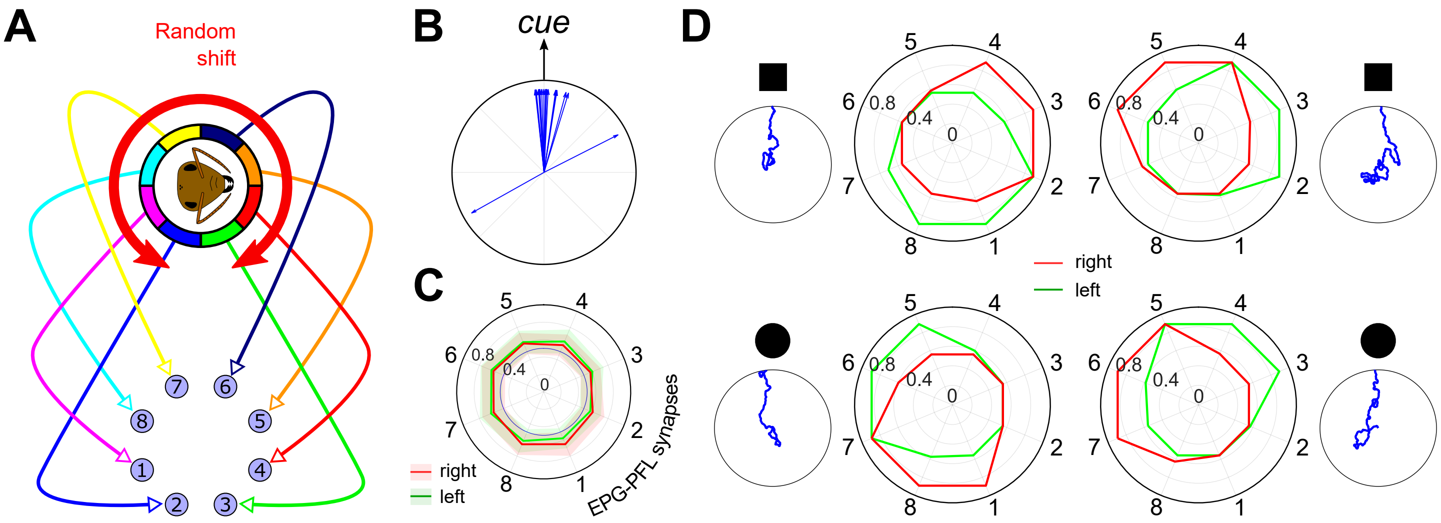

Fig D. Using an offset compass does not compromise the model function.
A. The connectivity between the visual units and the E-PGs has been shifted by a random value. The bump produced therefore expresses a constant offset different for every simulation.
B. Final direction vectors relative to the cue for 20 simulations.
C. Overall EPG-PFL synapse weights. The lines indicate the mean value for each EPG-PFL couple (red for the right side and green for the left) and the shaded area the standard mean deviation (s.e.m.).
D. 4 individual experiment paths and the associated EPG-PFL synapse weights generated during the simulation (red for the right side and green for the left).

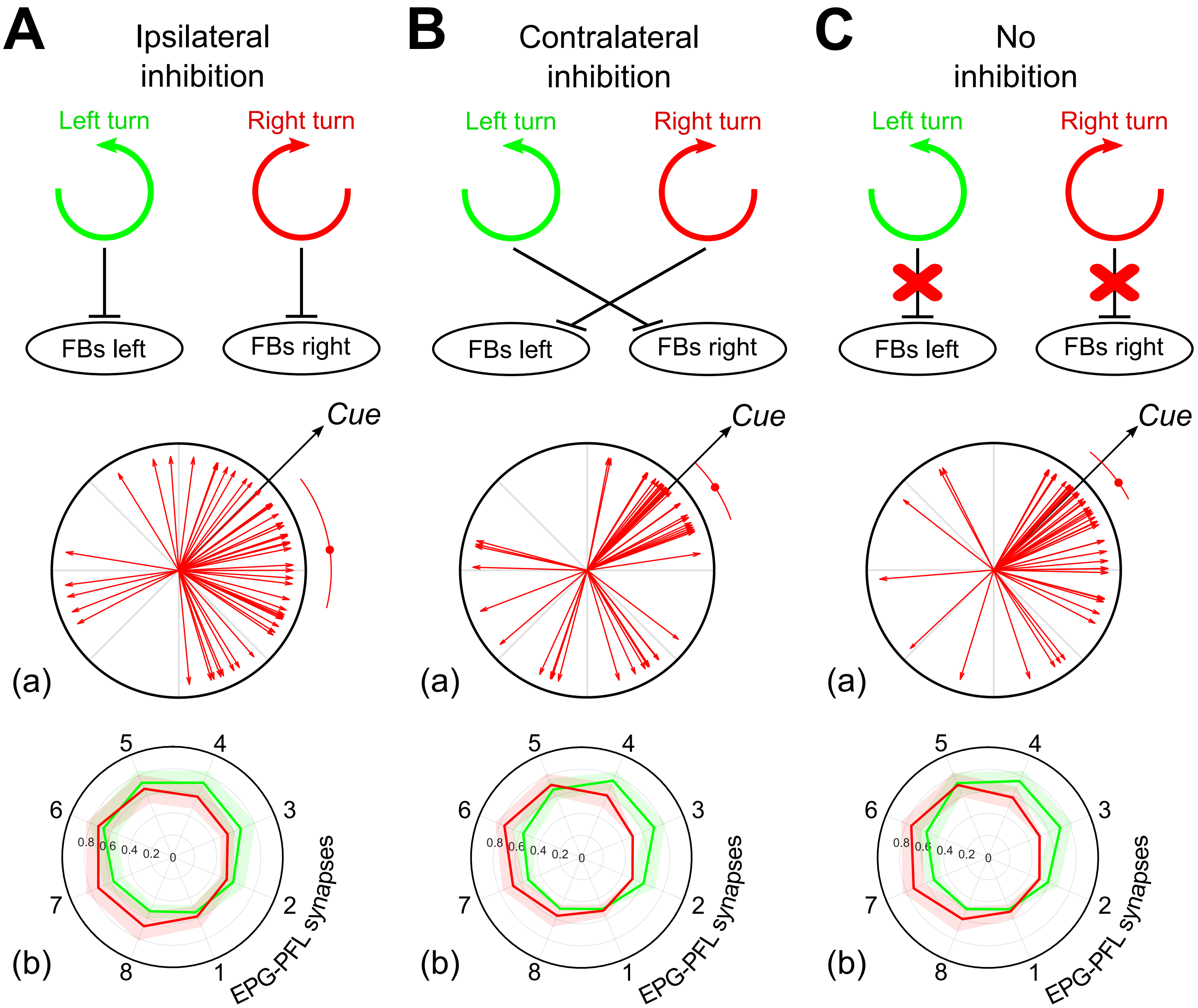

Fig E. Impact of the self motion integration in the model.
A-B-C. Model simulations with a combination of innate attraction and visual memory (MBs) as presented in figure 10A & B. (a) Final direction vectors for 50 simulations. The arc represent the median (dot) ± 95% C.I. obtained via bootstrap (rep = 10000). (b) Averaged right (red) and left (green) EPG-PFL synapse weights (shaded area: $\pm s.d.$) obtain during 50 simulations.
A. Ipsilateral inhibitory circuit from the self-motion signal to the FBns. This corresponds to the circuit presented in figure 6.
B. Contralateral inhibitory circuit from the self-motion signal to the FBns.
C. Circuit without any integration of the self-motion by the FBns layer.

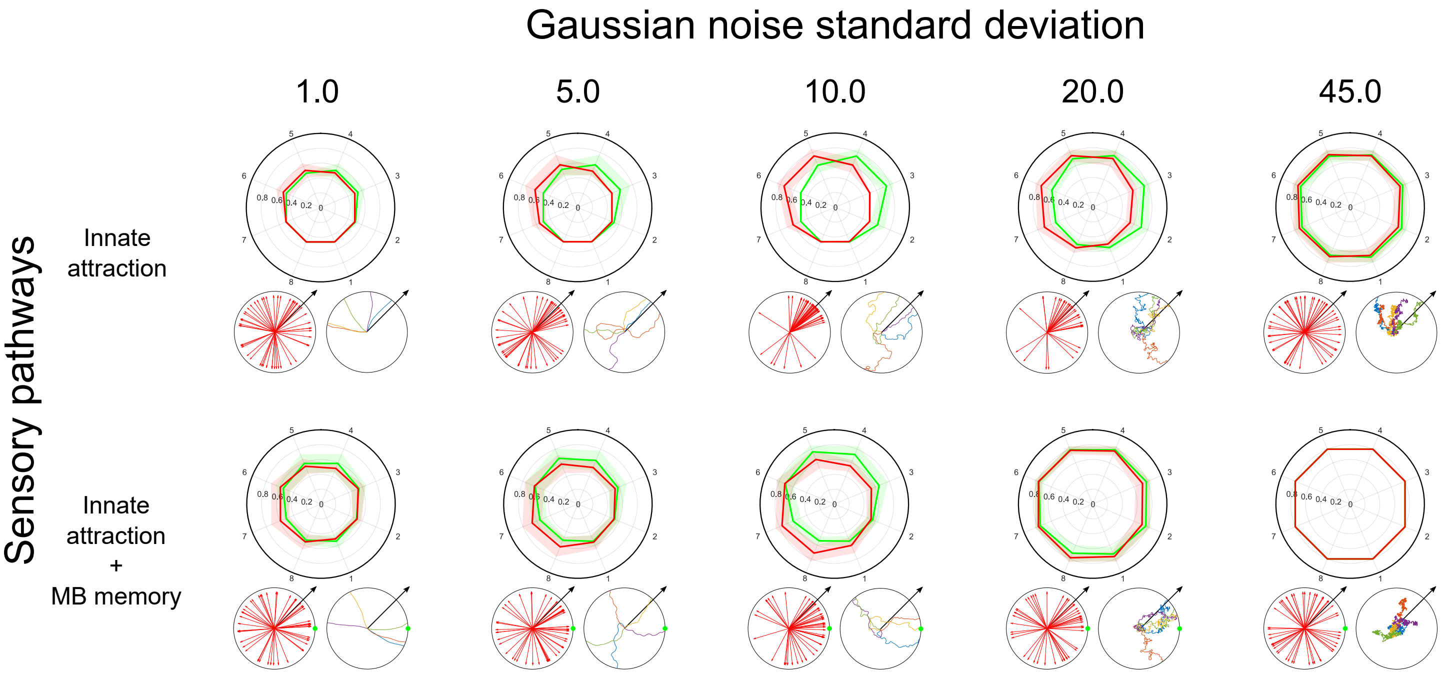

Fig F. Impact of the steering noise on the model performance.
The influence of the noise standard deviation applied to the steering is tested in the case of the innate attraction alone ($\omega_{MB} = 0$) and combined with the visual memory (ω_{Vin} and ω_{MB} set as in figure 10B).

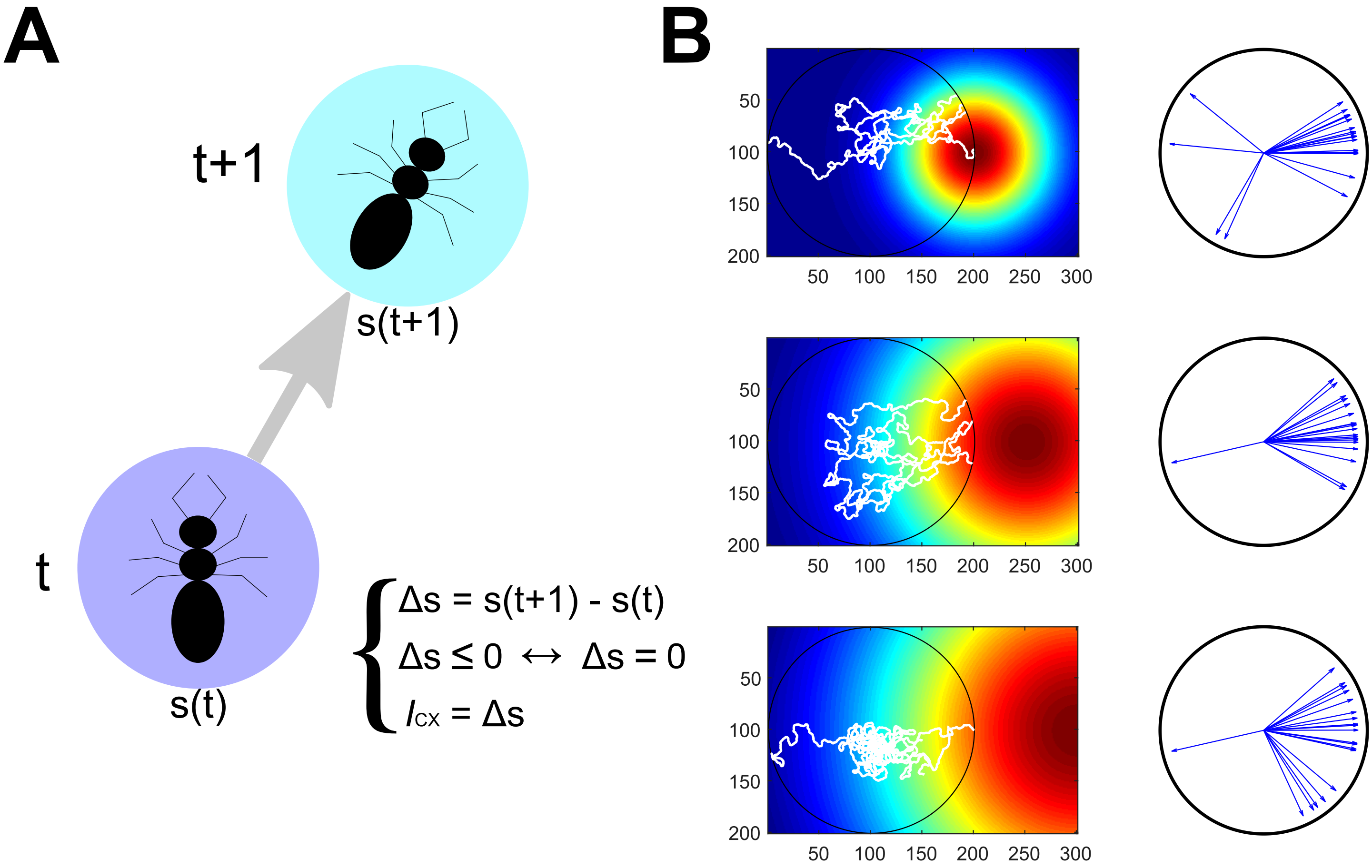

Fig G. Gradient ascent properties, potential for the olfactory pathway.
A. The reward signal (I_{CX}, input to the CX) is defined here as the difference (Δs) of the concentration in sensory input [$s(t)$]. Negative input, decrease of the concentration are not considered here ($\Delta s \leq 0 \;\leftrightarrow\; \Delta s = 0$). The value of Δ is multiplied by a free parameter ω_{Olf} to adapt roughly the amplitude to the same range as the visual pathways (innate and learned). During the simulations the visual compass is maintain, and keep it's function to modulate the EPG-PFL3 synaptic weights, thanks to a landmark randomly positioned around the arena.
B. Simulations with 3 different sources positioned at 100, 150 and 200 lu. from the arena centre creating a gaussian gradient (respectively s.d. = 50, 100 and 150 lu.). Left panels show the gradient shape and 5 paths example (white lines). Right panels show the final direction vectors of the full set of simulations (n = 20).

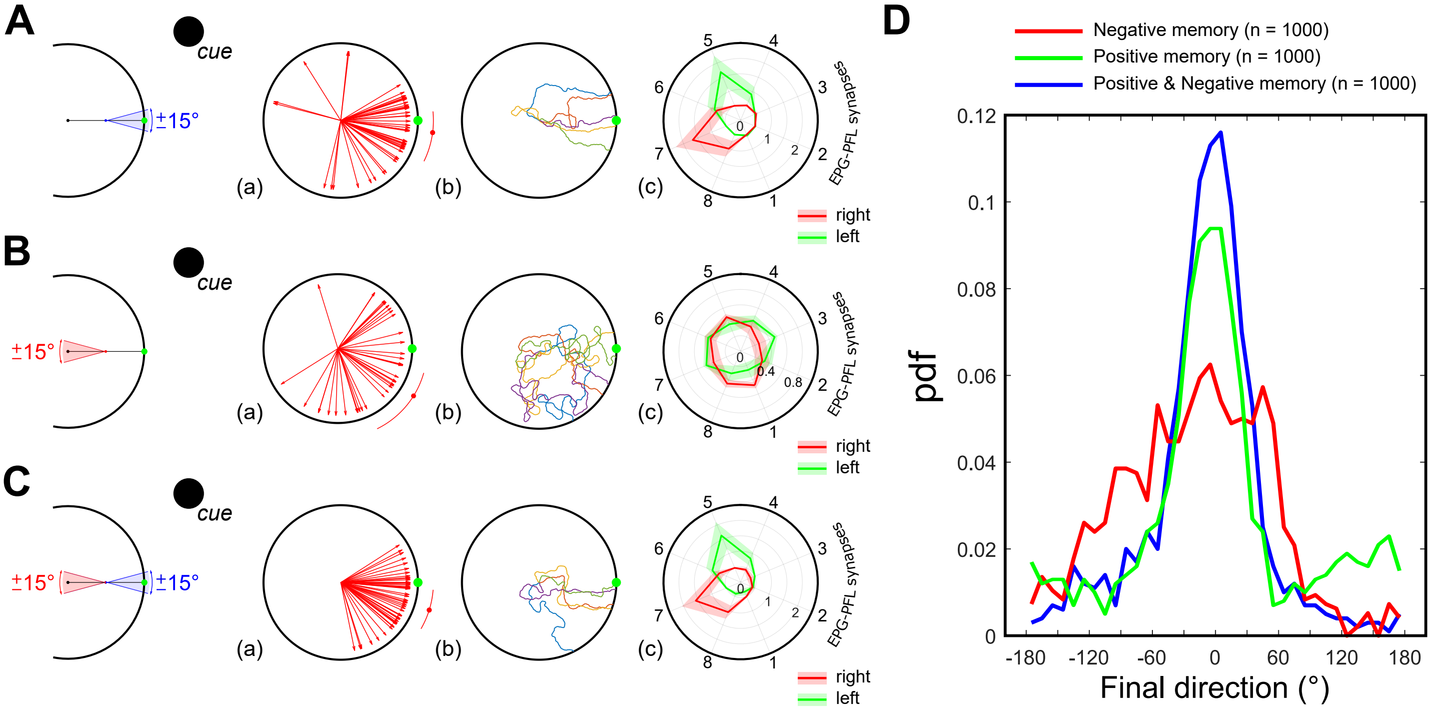

Fig H. Influence of an attractive-repulsive MB views memory on the model.
A-B-C. (a) Final direction vectors of 50 simulations. The red arc indicates the median (red dot) 95% C.I. obtained by bootstrap (n_{rep} = 10000). (b) 5 path examples. (c) Averaged right (red) and left (green) EPG-PFLs synapse weights (shaded area: $\pm s.d.$). For these simulations, in contrary to the other results presented in the paper, synapses weights were not capped to 0.8.
A. Simulations with attractive views memory only acquired facing (±15°, blue span) the feeder (green dot). This correspond to the same simulations as presented in figure 9.
B. Simulations with repulsive views memory only. MBON value is multiplied by -1 before connecting the FB neurons. Repulsive views are acquired in the same manner than the attractive one while facing $180° \pm 15°$ (red span) away from the feeder (green dot).
C. Simulations with both attractive and repulsive views memory. Each memory use the very same subset of KC neurons but is processed on a different MBON (positive for attraction, negative for repulsion).
D. Probability density function of the final directions for 1000 simulations in the 3 conditions (Attractive memory, positive memory and attractive & positive memory).

References

1. Stone T, Webb B, Adden A, Weddig NB, Honkanen A, Templin R, et al. An anatomically constrained model for path integration in the bee brain. Current Biology. 2017;27(20):3069–3085.e11. doi:10.1016/j.cub.2017.08.052.
