# Supplementary figures and images for: A unified mechanism for innate and learned visual landmark guidance in the insect central complex
Source: PLoS Comput Biol. 2021 Sep 23;17(9):e1009383. doi: 10.1371/journal.pcbi.1009383 (PMC8491911; doi:10.1371/journal.pcbi.1009383)

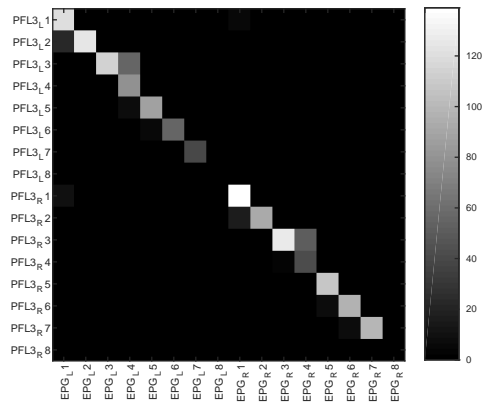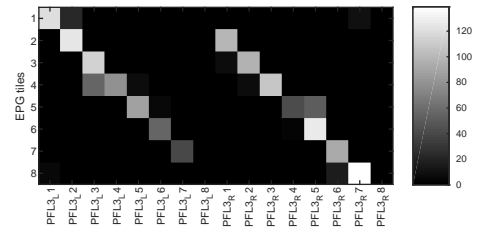

Supplement: S1 Table — S1_Table.csv: Table referencing the list of synaptic connection from EPGs to PFL3s. Presynaptic_Neuron: ID of the presynaptic neuron; PreN_ID: ID tag of the presynaptic neuron; Postsynaptic_Neuron: ID of the postsynaptic neuron; PpstN_ID: ID tag of the postsynaptic neuron; Nb_Synapses: Synapse count.EPG2PFL_connection.txt: txt version of the reference table. EPG2PFK_table_connections.csv: csv file containing the synaptic connection from EPGs to PFL3s as a cross table. TableSynapses.pdf: image plot representing the extracted cross table synaptic connection from EPGs to PFL3s. WeightCircular.pdf: circular plot conversion to the 1-on-1 EPG to PFL3 synaptic weights. (ZIP) [file pcbi.1009383.s002.zip › TableSynapses.pdf]

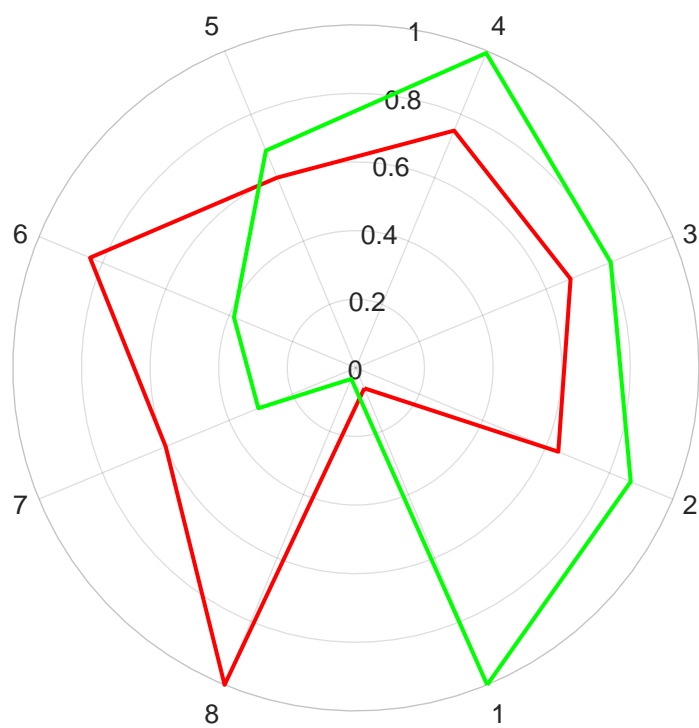

Supplement: S1 Table — S1_Table.csv: Table referencing the list of synaptic connection from EPGs to PFL3s. Presynaptic_Neuron: ID of the presynaptic neuron; PreN_ID: ID tag of the presynaptic neuron; Postsynaptic_Neuron: ID of the postsynaptic neuron; PpstN_ID: ID tag of the postsynaptic neuron; Nb_Synapses: Synapse count.EPG2PFL_connection.txt: txt version of the reference table. EPG2PFK_table_connections.csv: csv file containing the synaptic connection from EPGs to PFL3s as a cross table. TableSynapses.pdf: image plot representing the extracted cross table synaptic connection from EPGs to PFL3s. WeightCircular.pdf: circular plot conversion to the 1-on-1 EPG to PFL3 synaptic weights. (ZIP) [file pcbi.1009383.s002.zip › WeightCircular.pdf]
