# Supplementary material for: A unified mechanism for innate and learned visual landmark guidance in the insect central complex
Source: PLoS Comput Biol. 2021 Sep 23;17(9):e1009383. doi: 10.1371/journal.pcbi.1009383 (PMC8491911; doi:10.1371/journal.pcbi.1009383)
Supplement: Attachment — Submitted filename: PlosCB - Reviews.pdf [file pcbi.1009383.s003.pdf]

Comments to the Authors:
Please note here if the review is uploaded as an attachment.
Reviewer #1: Goulard and colleagues developed an insect central complex model to explain how the central complex might combine innate and learned orienting behaviors. It describes how non-navigational information, such as valence, could be used to modify synaptic weights to affect navigation behaviors.

The main contribution of this work is to provide a conceptual framework of how reward signal can be used to modify the synapses from head direction cells to motor command cells. The critical part is the introduction of the shifted modulation (figure 6), in columns, to generate stable orienting behaviors. This conceptual contribution is potentially important. However, there are a few concerns that need to be addressed.

1. [page 9] This work singularly picked PFL3 as its key player of the model, in which PFL3 neurons directly receive theinput from EPG neurons via synapses in the PB. On the other hand, the hypothetical set of neurons providing modulatory signal are modeled to be in the FB and they modify the synapses in the PB in shifted columns, an essential assumption of the model. I am not sure if this is anatomically (or even biologically) feasible or supported. Although the conceptual contribution of the model is still very interesting, based on anatomy, it is hard to imagine that PFL3 neurons (and the hypothesized FB neurons) are responsible for the proposed mechanism.

   The innate connectomic pattern between EPG and PFL3 seems ideal to induce oriented behaviour, nevertheless it is true that the involvement of PFL3 in the adaptive mechanism we propose is not supported by any direct evidence. However, the existence of connections from FBn to PFL3, with shifted columns, is supported (Stone et al., 2017, Lyu et al. (preprint)). Thus, this essential assumption is anatomically feasible, while it remains possible the exact mechanism or neural substrate may differ (see following comments).

2. There is no evidence that the synapses from EPG to PFL3 are plastic.

   We agree that the plasticity at the level of EPG to PFL3 synapses is not documented. Although we have assumed that this is the locus of the memory, we note that an alternative means of obtaining the same effect would be if the memory were accumulated in the FBn, analogously to the path integrator (PI) of honeybees (Stone et al., 2017) and these neurons either provide direct input, or modulate the EPG input, into PFL3. As we had already indicated in describing the adaptation mechanism, for the level of neural model we are using, these mechanisms are mathematically equivalent. We have added some text to make this point clearer and have also replicated the model results explicitly using this alternative (supplementary data, figure S.3). In the discussion, the use of the term “synaptic memory” or the mention of the “synaptic weight changes” has been replaced by the mention of the more generic “memory”, to avoid confusion about our claims.

3. Related to above: The synapse data from EM in Figure 4 shows strong symmetrical bias in left and right connections. It is not clear if this kind of strong anatomical structure can be achieved in a short-term implied in this work. If synapses are plastic in a short-term, the actual synaptic weight might be modified not in the number of synapses but rather in some other mechanisms (such as the postsynaptic receptor concentration/distribution). For example, the synapses between Ring neurons and EPG neurons have been hypothesized to be plastic (Kim et al., 2019; Fisher et al., 2019), yet the EM data shows nearly homogeneous number of synapses between them (Hulse et al., 2020).

   We did not intend to claim that the short-term plasticity correlates to a change in the number of synapses; any form of plasticity such as pre- or post- synaptic receptor concentration change could be a possibility. Again, at the level of modelling we use, the alteration is simply a ‘synaptic weight change’. However, it seemed conceptually simpler to start from Rayshbuskyi et al.’s approach, using connectomic data to identify a `weighting’ asymmetry that could support steering, and then generalise this idea first to steering in arbitrary directions and then to steering in a direction related to experienced reward.
   - We have clarified our absence of claim on a particular memory mechanism in the presentation of the model (l.185).
   - We have added a paragraph in the discussion to address the short-term plasticity of R-neurons in the CX that could match our model without showing a modification of the connectome (l.338).

4. From eq (4), the synaptic weights may not be stable if the simulation is continued over multiple scenarios. For example, if the reward mask in the front moves slowly counter clockwise over time, while the plasticity is ON,

then the entire weight would be eventually saturated during the simulation. In other words, the synaptic update is only one-way, which, without a ‘forgetting’ mechanism, may result in unstable agent behaviors.

We only include a one-way update of the synaptic weight/memory in the model as the paradigm we used is simple enough not to produce complete saturation and/or instability in the model. Complete saturation would only happen if the agent was allowed to explore 360° directions around the cue, however, limited to our simulated arena with the cue placed far outside, the model would still produce consistent memory/synaptic weight even during far longer (potentially infinite) explorations. However, a forgetting mechanism would be crucial to allow more complex route following. We believe that adding a more complete dynamics, including forgetting, could act as a filter similar to the mechanism in Wystrach & al (2019) which they show can be used for route following. In their preprint, Wystrach & al (2019), use the unilateral MB memory that is integrated by the FB to modulate the amplitude a +/-45° rotated copy of the compass. They use a temporal window to filter the signal received by the FB from the MB, which could smooth the discrepancy that exist in the MB. This will allow us to investigate the potential of the model to grasp more complex navigation behaviour in the future. A paragraph has been added to the discussion to mention the absence of forgetting mechanisms, and the consequent limitations, in the present model (l.443).

5. line 131-132: It says the connection from EPG to PFL3 is inhibitory since EPGs are cholinergic, which means excitatory. As the polarity of the connection from EPG to PFL3 is critical for the model behavior, this discrepancy needs to be clarified.

   We agree that this discrepancy is not justified, it is an artefact from the inspiration from Stone et al. model where TB1 to CPU1a/CPU4 are inhibitory. We therefore have reversed the EPG to PFL3 connection to be excitatory.This can be easily compensated by altering the polarity of the mapping from left and right PFL activity to Left turn *vs* Right turn, which was arbitrarily set. Consequently, the model behaviour is unchanged. We added the equation of the change of steering in the description of the model (eq. 4).

6. The description of figure 6 and the eq (4) is arguably the most important part of the paper, yet it was not easy tograsp the key idea. I strongly suggest more polishing.

   We added a panel in figure 6 to represent the synaptic modulation, and a sentence in the description of the consequence of equation 6 (formerly eq. 4) to clarify the operant component of the mechanism. We hope this makes the key idea clearer.

Minor comments

- A lot of subpanels of figures are not referenced in the main text, which may leave readers confused. Figure 1 is not even mentioned in the main text.

  Done

- Line 17: Ref [17] does not seem a right one to cite. Taube et al 1990 that found HDCs seems more appropriate.

  Thank you for pointing this out, we have made this change.

- The edge index used to calculate the output of visual neurons is hard to understand its behavior and meaning. Thetext says that ‘S_left’ and other three ‘S’s are the summed intensities of the corresponding half. Then what does $I_i = 0$ (and $I_i = 1$) mean? In my calculation, I_i would be 1, only if the bright pixels are only on the left top corner, no matter how the edge shapes. On the other hand, if there is a vertical edge at the center of the scene, $I_i = 0.75$. Please clarify what I_i really means.

  The index I_i is designed to be greater when observing a corner (I_i close to 1, independently of its shape) than a single edge (I_i close to 0.75). We added a sentence at the end of the paragraph to explain it (l.84).

- Table 1 in the supplementary info needs to be sorted.

  Done

- Table 1 in the supp info and Figure 4 shows 7 PFLs on each side, whereas the text says there are 12 PFLs for eachside. Is there a reason for this discrepancy?

  It is indeed a mistake in the text, it has been modified.

- line 266-276: It is not clear whether the modification of the synapses from EPG to PFL3 happens after the lesion, orthe lesion was performed after the learning. I assume the former, since the reward signal, which is used to modify the synaptic weight, is affected by the lesion; It needs to be clarified.

  We have added the timeline of the simulations involving the MB (Learning phase [MB update] -> Test [CX update]) in figure 9 and modified the text to match the denominations learning phase and test.

- line 378-386: The relationship between Neuser at al. and this work is not clearly described in the discussion. (Is itfuture work? Or can this model replicate it? Or what kind of mechanisms are necessary to replicate it?)

  We believe the Neuser experiments are an example of the interplay between taxis and short-term/working memory behaviour in the CX, helping to justify the involvement of the CX in innate behaviour. However, we recognize that the model would need several modifications/additions to reproduce a similar behaviour. We therefore have removed the figure describing this paradigm to avoid any confusion about what has been done and clarify in the text how it is related to the current work (l.432).

Reviewer #2: The authors build a neuroanatomical model including head direction neurons in the central complex to produce observed innate attraction to visual clues. They integrate the mushroom bodies into the model, which they used to encode visual memory and to enable memory-based directional navigation in a simulated agent. The core idea of the model is interesting and relies on the connectivity from EPG to PFL3s neurons, which the authors found to be heterogeneous from the fly connectome (Figure 4b). Since PFL3s neurons output to previously reported DaN2 neurons involved in steering control [Wilson lab, biorxiv (2020)], the authors argue that these heterogeneous connections from EPG to PFL3 produce fixation towards a desired direction.
One issue that is still puzzling is the assumption that EPGs are inhibitory to PFL3, which seems not to be supported by experimental evidence. This also seems to be at odds with another assumption: that EPG neurons are excitatory to PEN and PEG neurons.
The polarity of EPG neurons in the model should therefore be clarified. It seems that assuming EPG to be excitatory to PFL3 neurons would result in the agent going away from the visual clue.
A second issue that would be good to improve is some biological justification for the plasticity, since these rules are not standard.
Comments:

- Line 132: “EPG are reported to form cholinergic synapses”: citation missing.

  Citation added.

- Line 132: The authors assumed in equation (2) that EPG provide excitatory input to PEG and PEN neurons, while now they assume EPG neurons provide inhibitory input to PFL3 neurons. EPG are reported as excitatory for example in [Franconville, R. …, Jarayman, V. Elife (2018). Elife], where functional connections involving presynaptic EPG neurons are always excitatory.

  We agree, see response to point 5 of Reviewer 1.

- Line 132: Missing equation for activity of PFL3 neurons

  Equation 3 added.

- Line 145: If the model assumes excitatory connections between EPG and PFL3, then the agent would not beattracted to the stimulus but would avoid it and position the stimulus in the back.

  Yes, but an arbitrary change in the left/right effect of PFL activity can restore the desired polarity. See response to point 5 of Reviewer 1.

- Figure 5B: The direction of the agent in the absence of Gaussian noise is fully determined by the connectivity and the preferred direction will be defined by the intersection point between the left and right connectivity profiles of

EPGPFL (intersection between green and red line in Figure 5B, top left). It would be helpful, if the authors explained this in more detail, and then added the noise that produces stochastic trajectories.

We have clarified in the description of the model that the general direction taken by the agent is determined by the intersection between the two sets (left and right) of EPG-PFL3 synapse weights (l.155). We also emphasised the role of gaussian noise to generate the initial exploration is crucial to acquire the CX memory in the model (l.228)

Line 165: Some neurons in the FB perform column-shift operations (for example, see hAB neurons in [Cheng L. …,Maimon, G. Biorxiv (2020)]). Why not include a population from the fly connectome that matches the population 'FB' that the authors introduce?

We have added in the discussion two sentences introducing the P-F3N2d and P-F3N2v populations (Franconville et al., 2018) as hypothetical targeted populations as they correspond to the CPU4a/b populations that underly the path integration in Stone et al., 2017. These two populations present the advantage of forming connection from the FBs to the PB as well as with the noduli, which could support the integration of the self-motion in the model.

- Equation (4) is an unusual plasticity rule. It has 2 modulatory signals that have to coincide in time: activity of FB(t) neurons and a reward signal Rew(t). It would be helpful to add more biological justification for this rule.

We agree that this rule may lack biological justification. As discussed in our answer to reviewer 1, it is not essential to the concept of our model that the influence of the FBn on the PFL takes exactly this form, i.e., combining with the reward to alter the EPG-PFL weights, and an alternative would be that the reward gates the accumulation of activity in the FBn which is then combined with the EPG input at the PFL. As noted in our response to reviewer 1, we have implemented this alternative and show the results are the same (figure S.3). We have added some sentences in the presentation of the learning rule to clarify that the rule is used for conceptual convenience rather than being motivated directly by biological evidence (l.199).

- Line 204: When the model uses negative input, the performance increases. Missing a qualitative measurement for performance to compare between different inputs.

We have added a subpanel (b) in figure 7, instead of the previous heatmap (redundant with other panels), with the probability density function of the final direction vectors relative to the cue orientation during the simulations. When a negative reward is combined with the positive one the model shows an increase by 10% of final direction to the landmark.

- Line 205 (Figure instead of 'igure')

Done

- Figure 7 top row: What is the irregular black semitransparent shadow? Is it the mask? If so, why does it have such an irregular shape?

The shape represented a random object, used in figure 2 to show the visual processing. We remove the object displayed behind the filter in figure 7 to avoid confusion.

- Equation (7) does not include time dependence as the previous equations.

Added in equation (9).

- Table 1 seems unnecessary. Why not write in equation (7) the weights w_i^KC-MBON in terms of the activities ofDAN and KC:

w_i^KC-MBON = w_i^KC-MBON – 0.2 * DAN * KC
Still this synaptic rule needs biological justification.

We believe this learning rule is quite common in MB models and is supported by neurobiological observation. It has been inherited from Ardin et al, 2016. We believe presenting it as a table make it easier to grasp the associative extent of this rule.

- Line 247: During the 100 first steps of the simulation, the agent scans the environment from -15 to 15 degrees. How is this exactly done? During these 100 first steps, are the DAN neurons set manually to 1 so they create the memory?

  The agent scans the environment with a 0.3°/step rotation starting 15° on the right of the feeder orientation (supposed known by the agent during the learning phase) and finishing 15° on the left. We consider this to be an intentional 'learning action' by the agent comparable to the 'turn back and look' behaviour that has been described in ants departing from a location in which they were rewarded (Nicholson et al 1999). The paragraph has been rewritten to make this clearer.

- In that case this is a training phase. If after the 100 first steps, the agent is let alone in the environment with activity of DAN neurons set to 0, that is a test phase. Need to clarify (and maybe add a figure) about the timeline and different phases during the simulation.

  It is indeed a learning phase (during which DAN = 1) separated from the test phase (during which DAN = 0). The simulation sequence has been added to figure 9B, and the text has been modified to match the denomination learning phase and test.

- Line 252: Needs more justification and a mathematical expression of how the input signal from MB to the CX is ignored in the model. Add to Methods or supplementary information.

  The output of the MBON is simply thresholded at 0.25 (MBON = 0 when lower than 0.25). We modified the sentence to make it clearer.

- Line 265: What is the evidence that the two MB structures (located in each hemisphere) get visual input from both eyes?

  The detailed connection pattern is not known but there exists some behavioural evidence for crossing over (eye covering experiments). For the model purpose, it is the most parsimonious assumption as each MB should have a similar outcome.

- Discussion could also be shorter, some of the text could go to the introduction, for example most of section 4.3

  It was not clear to us which parts of the discussion should be made shorter, and we feel it is more useful to discuss the potential advantages of CX involvement in innate steering after we have shown the innate steering preference emerges from the CX connectivity data. We have tried to edit some of the text to be more succinct.

Reviewer #3: This is an interesting and timely theoretical study in the field of spatial navigation. The authors have used the most up-to-date experimental and anatomical data to construct a nice model of the compass-guided steering system in the insect brain. The basic steering model (Figure 5) is similar to that of Rayshubskiy et al. (ref. 30). The novel aspect of this manuscript is primarily the further extension of that basic model, as shown in Figures 6-8. In this extension, the authors show how the system can learn to adopt a new heading goal associated with some rewarding cue. This learning process relies on a specific hypothetical class of fan-shaped body neurons (called here FBn) which combine heading signals and steering signals. The output of these FBn neurons is used to drive associative plasticity at compass neuron output synapses. This in turn changes the way that the compass is "read out" by the steering system.

Major points:

1. 131 – “The synapses between EPGs and PFL3s are represented as inhibitory (Figure 5A) as EPGs are reported toform cholinergic synapses.” – Cholinergic synapses are excitatory, not inhibitory. Thus the synapses between EPGs and PFL3s should be excitatory. I think this will flip the stable fixed point of the model to the opposite side of the ellipsoid body. I think it will also necessitate an inversion of the learning rule in Equation 4.

   We agree, see response to point 5 of Reviewer 1.

2. In addition to the sign inversion mentioned above, I think there must be another sign inversion somewhere in the model. This is because the stable fixed point of this system centers the EPG bump on glomeruli 5R/5L (as shown by Rayshubskiy et al.), but when EPG-to-PFL3 synapses are set to be excitatory, this should flip the stable fixed point of the model. Thus, I think some other sign inversion must be made to restore the correct fixed point. I can’t figure out exactly what this sign inversion is, but the authors may be able to determine this. It may have to with how heading direction changes are mapped onto the EPG array.

   The polarity of the Left-Right PFL3 comparison (which was arbitrary in the first place) has been reversed to compensate the modification of the EPG-to-PFL3 synapses to excitatory.

3. A key feature of the model is that EPG synapses onto PFL3_left and PFL3_right neurons can be differentially strengthened during right- versus leftward turns. The source of this direction-selective turning-related input is some abstract cell type in the FB (“FBn”). I understand that this is a conceptual model, and the specific identity of the cell in question does not really matter for the larger conceptual point. But if the model has any relevance to the brain, then there should be at least some conceivable pathway where this direction-selective turning-related input could originate. The authors suggest this input comes from the noduli, which seems like a good guess. If so, they should be able to show that a given PFL3 neurons receives mainly input from either the left-nodulus OR right-nodulus. Of course, these connections may be indirect, but still it should be possible to show that there is differential indirect anatomical input from the left-nodulus or right-nodulus onto any given PFL3 neuron. I am not asking the authors to do any experiments – I am just asking them to look at the connectome and point out a pathway that could make this idea plausible.

   The recent observation of noduli input to a pathway projecting from EPGs to the hAB neurons in the FB could support the influence of self-motion into FB neuron populations. In addition, we introduced in the discussion the neuron populations P-F3N2d and P-F3N2v (that correspond to CPU4a/b, Stone et al., 2017). However, the inhibitory pathway is still arbitrary. This choice has been made to create an imprint/a memory, by the CX, of the active behaviour during the exploration that fit well an operant mechanism.

4. I am confused as to whether the FBn inputs to PFL3 neurons (Figure 6) are active all the time (during all navigation), or only during learning. If they are active all the time, wouldn’t they tend to truncate and reverse any turning behavior – because rightward turns cause an immediate withdrawal of excitatory drive to the PFL3 neurons that drive rightward turning, and an increase in excitatory drive to the PFL3 neurons that drive leftward turning? If FBn inputs are postulated to be inactive except during learning, then how is this thought to work, and how is learning separated from navigation per se?

   The FBns are active during the whole simulations’ duration (except during the learning phase when MB are involved). This could lead to infinite increase of the weights if the simulations were not limited to a restricted space. It would lead to a homogeneous pattern only if exploration of the environment all around the cue was allowed (considering it is the only cue accessible to maintain the compass function). In the case of an infinite distance of the cue (if the sun is used for example), it would increase infinitely the weights strength but would maintain a pattern allowing a correctly oriented behaviour as the direction taken depends on the comparison of both sides and not on their actual values. In addition, the output of the model (before addition of the noise) is constrained to mimic neurological/physical limitations. Equation 5 has been added to clarify the calculation of the steering output of the model.

Minor points:

5. 94 – “mimicking bar fixation experiments in Drosophila” – Bar fixation does not require the central complex (see Green et al. 2019 ref. 18 and Giraldo et al. 2018 ref 19). Therefore this sentence is potentially confusing and should be omitted. As noted in the next sentence, fixation is not the task which is being modeled here anyhow; rather, the task is to hold the visual cue at an arbitrary angle.

Modified to cite the bar fixation experiments only as the paradigm in which the compass has been first observed and described.

6. Figure 3B, regarding the retinotopic mapping from visual neurons to EPG neurons: this is actually a likely accurate description of the functional mapping from Ring neurons to EPG neurons. See for example Kim et al. Figure 2e (ref 57) and Fisher et al. 2019 Nature Figure 5g (https://doi.org/10.1038/s41586-019-1772-4).

   It is hard to confirm a correspondence between our visual to EPGs mapping and Ring neurons to EPGs as (1) Any Ring neuron project to all EPGs (2) this mapping is subject to plasticity mechanisms (Fisher et al., 2019). However, we believe we show that the behaviour of our model is independent of such specific mapping (Figure S.3).

7. Figure 3C: It should be noted explicitly in the legend that the compass is here depicted as viewed from the posterior side of the brain. If the compass is viewed from the posterior side, then the bump moves counterclockwise as the fly turns right, and vice versa ((Turner-Evans et al., 2017).

   Done

8. 136 – "More specifcally, the specific weights/connections pattern (Figure 5B) that frames the EB bump to the front of the visual field would suffice to generate an innate attraction to the conspicuous landmark." – See major point 1 above. This should be reversed: front->back (or attraction->repulsion).

   The Left-Right comparison has been reversed (as it was arbitrarily set initially) and kept its consistency with the front/attraction.

9. 159 – "in our case the motion input corresponds to left and right rotations rather than translation" – It seems worth noting here that sideways translation is highly correlated with rotation, and so it would be mathematically impossible to be highly correlated with one but not the other.

   As the model does not generate any sideways motion, we did not consider any kind of correlation between translation and rotation. To avoid any confusion the sentence has been simplified and the mention of translation has been removed.

10. 168 – "primarily motivated by the permanent modification that could define an innate preference for vertical bars". The innate preference to fixate (center, approach, etc.) a vertical bar does not require the central complex (see Green et al. 2019 ref. 18 and Giraldo et al. 2018 ref 19).

    The paragraph has been modified. The motivation to design the memory in the model as a synaptic modulation is to keep the two sections consistent: the observation of the connectome and the mechanism proposed to ensure the behaviour plasticity.

11. Re: section 3.7, it seems relevant to note that there are several MBONs that make an unusually large number of synapses onto FB tangential cells (Li et al. eLife 2020, https://elifesciences.org/articles/62576).

    Indeed, it is a nice evidence for the influence of the MB on the oriented behaviour in the CX. Added.

12. It would be useful to provide Table 1 in machine-readable format.
    Table 1 has been submitted as well as a csv format file.
